# Preference of carbon absorption determines the competitive ability of algae along atmospheric CO_2_ concentration

**DOI:** 10.1002/ece3.9079

**Published:** 2022-07-11

**Authors:** Qing Shi Zhou, Yang Gao, Jing Ming Hou, Tian Wang, Long Tang

**Affiliations:** ^1^ State Key Laboratory of Eco‐hydraulics in Northwest Arid Region Xi'an University of Technology Xi'an China; ^2^ State Key Laboratory of Eco‐hydraulics in Northwest Arid Region, Institute of Water Resources and Hydro‐electric Engineering Xi'an University of Technology Xi'an China; ^3^ School of Human Settlements and Civil Engineering Xi'an Jiaotong University Xi'an China

**Keywords:** affinity, algae, bicarbonate, carbon absorption, carbon dioxide, competition, flux rate

## Abstract

Although many studies have focused on the effects of elevated atmospheric CO_2_ on algal growth, few of them have demonstrated how CO_2_ interacts with carbon absorption capacity to determine the algal competition at the population level. We conducted a pairwise competition experiment of *Phormidium* sp., *Scenedesmus quadricauda*, *Chlorella vulgaris* and *Synedra ulna*. The results showed that when the CO_2_ concentration increased from 400 to 760 ppm, the competitiveness of *S. quadricauda* increased, the competitiveness of *Phormidium* sp. and *C. vulgaris* decreased, and the competitiveness of *S. ulna* was always the lowest. We constructed a model to explore whether interspecific differences in affinity and flux rate for CO_2_ and HCO_3_^−^ could explain changes in competitiveness between algae species along the gradient of atmospheric CO_2_ concentration. Affinity and flux rates are the capture capacity and transport capacity of substrate respectively, and are inversely proportional to each other. The simulation results showed that, when the atmospheric CO_2_ concentration was low, species with high affinity for both CO_2_ and HCO_3_^−^ (HCHH) had the highest competitiveness, followed by the species with high affinity for CO_2_ and low affinity for HCO_3_^−^ (HCLH), the species with low affinity for CO_2_ and high affinity for HCO_3_^−^ (LCHH) and the species with low affinity for both CO_2_ and HCO_3_^−^ (LCLH); when the CO_2_ concentration was high, the species were ranked according to the competitive ability: LCHH > LCLH > HCHH > HCLH. Thus, low resource concentration is beneficial to the growth and reproduction of algae with high affinity. With the increase in atmospheric CO_2_ concentration, the competitive advantage changed from HCHH species to LCHH species. These results indicate the important species types contributing to water bloom under the background of increasing global atmospheric CO_2_, highlighting the importance of carbon absorption characteristics in understanding, predicting and regulating population dynamics and community composition of algae.

## Graphical abstract

The differences in affinity and flux rates of CO2 and HCO3− could explain the changes of competitive advantage between algae species along the gradient of atmospheric CO2 concentration. With the increase of atmospheric CO2 concentration, the competitive advantage changed from species with high affinity for both CO2 and HCO3− to species with high affinity for CO2 and low affinity for HCO3−.

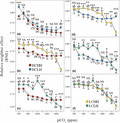

## 1 INTRODUCTION

With the increase of atmospheric CO_2_, many plant ecologists have taken an experimental or modeling approach to identify the growth, reproduction and distribution of algae along the gradient of CO_2_ concentration (Bolton & Stoll, 2013; Brown et al., 2019; Hammer et al., 2019; Low‐Décarie et al., 2011). However, few studies have shown how the relative concentration of CO_2_ and HCO_3_^−^ in water affect the algal growth and competitive advantage at a population level (Beardall & Raven, 2017; Li et al., 2015; Low‐DÉCarie et al., 2011; Ma et al., 2019; Pardew et al., 2018; Sandrini et al., 2016; Van de Waal et al., 2011; Verspagen et al., 2014). Some CO_2_ in the water is hydrolyzed to HCO_3_^−^, which changes the pH value of water, and then affects the relative concentration of CO_2_ and HCO_3_^−^ in the water along the gradient of atmospheric CO_2_ concentration. The preference for CO_2_ and HCO_3_^−^ is different between algal species due to evolution (Litchman et al., 2015; Schippers, Lurling, et al., 2004a; Schippers, Mooij, et al., 2004b). Therefore, studying the changes in the relative concentration of CO_2_ and HCO_3_^−^ in water along the gradient of atmospheric CO_2_ concentration and the effect of these changes on algal growth and interspecific competition ability is an important perspective for understanding and predicting the changes in population dynamics and community composition of algae under the background of increasing global atmospheric CO_2_, and therefore an basis for maintaining the health of an aquatic ecosystem.

The carbon absorption of algae includes the capture and transport of CO_2_ and HCO_3_^−^ (Hammer et al., 2019; Xiao et al., 2017). The affinity and flux rates of substrate CO_2_ and HCO_3_^−^ vary among algal species (Reinfelder, 2011; Stojkovic et al., 2013). Affinity refers to the ability of the binding site on the transporter to capture the substrate, while flux rate refers to the maximum transport capacity of the transporter when the binding site is saturated (Lines & Beardall, 2018; Sandrini et al., 2014). Many studies have shown that high affinity is usually accompanied by a low flux rate (Hepburn et al., 2011; Reinfelder, 2011; Stojkovic et al., 2013; Tortell, 2000). When the substrate concentration is low, the species with high affinity perform better; in contrast, when the substrate concentration is high, the species with a high flux rate perform relatively better (Lines & Beardall, 2018; Reinfelder, 2011; Sandrini et al., 2014). Therefore, the two metrics are effectly capturing different aspects of carbon absorption and, subsequently, should profoundly impact the growth and competition of algae along the atmospheric CO_2_ gradient.

Based on previous studies, we predict that when the CO_2_ concentration in the atmosphere is low, both CO_2_ and HCO_3_^−^ concentrations in water are low, which is favorable for the growth, reproduction of algae with high affinity for both CO_2_ and HCO_3_^−^, and such species would be competitive dominant (Schippers, Lurling, et al., 2004a; Schippers, Mooij, et al., 2004b). When the atmospheric CO_2_ concentration is high, the pH of the water is low, and the water has relatively more CO_2_ and less HCO_3_^−^ (Brown et al., 2019; Hasler et al., 2016). In this way, as atmospheric CO_2_ continues to increase, the increase rate of CO_2_ in water increases, while the increase rate of HCO_3_^−^ decreases, and the content of HCO_3_^−^ may even decrease. Therefore, algae with low affinity for CO_2_ and high affinity for HCO_3_^−^ would be competitive dominant when atmospheric CO_2_ continued to increase.

In this study, a pairwise competition experiment was conducted to investigate changes in the growth and competitive advantage of four species of algae (*Phormidium* sp., *Scenedesmus quadricauda, Chlorella vulgaris* and *Synedra ulna*) when the atmospheric CO_2_ concentration increased from 400 ppm to 760 ppm. A model was developed to explore whether interspecific differences in affinity and flux rate for CO_2_ and HCO_3_^−^ between algal species could explain these changes. The purpose is to highlight the importance of carbon preference in algal growth, reproduction, and competition along atmospheric CO_2_ concentrations, contributing to our understanding of algal population dynamics and community composition along environmental gradients and providing a direction to predict bloom causing species in the context of increasing global atmospheric CO_2_.

## 2 MATERIALS AND METHODS

### 2.1 Investigation

To study the response of algal growth and competition to atmospheric CO_2_ concentration, a three‐factor design with 3 replications was used. The factor species were cyanobacteria, *Phormidium* sp.; green algae, *Scenedesmus quadricauda* and *Chlorella vulgaris*; diatoms, *Synedra ulna*. Culture treatments were monoculture and mixture of two species, and therefore the treatments of monoculture and mixture were 4 and 6. The atmospheric CO_2_ concentration was 400 ppm (“low CO_2_”) or 760 ppm (“high CO_2_”). All four kinds of algae were purchased from the Freshwater Algae Culture Collection at the Institute of Hydrobiology (http://algae.ihb.ac.cn/), and then cultivated in a biochemical incubator (BJPX‐150, Biobase, China) to the required amount (> 10^7^ Cells/L), used as the original algae sample. The medium was configured according to the composition and concentration of BG11. 400 ml of medium was placed in a 500 ml beaker, and the inoculation density of each algal sample in each beaker was 10^6^ Cells/L. A total of 420 such beakers were divided into 10 groups of 42 beakers each, which were 4 groups of monoculture species and 6 groups of mixture species.

Half of each group of samples (21 samples) were randomly selected and placed in an artificial climate chamber with 400 ppm CO_2_ gas, and the other half was placed in the artificial climate chamber with 760 ppm CO_2_. The artificial climate chamber was connected with a CO_2_ cylinder, which can adjust the indoor atmospheric CO_2_ level to the set concentration. Cultures were stirred 3 times a day. Three samples from each group in each climate chamber were randomly selected to measure algal density and water quality every 3 days. Other algae samples continued to grow. In this way, each sample was independent. A monoculture treatment was used as a control to study the competitive ability. For example, the number of individuals in monoculture treatment of *Phormidium* sp., compared to the number of individuals of *Phormidium sp*. in mixture with *S. quadricauda*, *C. vulgaris* and *S. ulna*, respectively. Such measurements were made seven times in total.

0.1 ml solution was taken from each sample after fully stirred, and then poured into a 0.1 ml, 20 mm × 20 mm counting chamber. The algae density is calculated by the equation

(1) $$N=n\times \frac{A}{A_{c}\times V}\;$$

where *N* is the algal density; *n* is the counted number of algae; *A* is the area of counting chamber; *Ac* is the area of visual field × number of visual fields; and *V* is the volume of counting chamber.

After the population density in monoculture and mixture experiments were calculated, the competitive ability of each species was calculated by relative neighbor effect (RNE). This method was proposed by Markham and Chanway for the calculation of competition intensity among individuals of higher plants (Markham & Chanway, 1996). After redefining the parameters, the competitive advantage among algae species was estimated from the equation:

(2) $$\mathrm{RNE}=\frac{P_{-N}-P_{+N}}{X}$$

where *P* is the algal density in the presence (*+N*) and absence (−*N*) of neighbors; *x* is *P*_−*N*_ when *P*_−*N*_ is greater than *P*_*+N*_; and *x* is *P*_*+N*_ when *P*_*+N*_ is greater than *P*_−*N*_. The *RNE* is positive when the interaction is competitive, and a relatively low *RNE* indicates competitive dominance.

We used an analysis of variance (ANOVA) followed by Tukey's honestly significant difference (HSD) test to test the effects of CO_2_ and species on the RNE value. An ANOVA followed by Tukey's HSD test was used to test the effects of measurement time (length of growth time), interspecific interaction, CO_2_, and species on growth rate of algae. The significance level was set at 0.05. These analyses were performed using SPSS 22.0 (IBM, USA).

### 2.2 Model

Our results and previous studies suggested that different species responded differently to increased atmospheric CO_2_ concentrations, even though they belonged to the same taxon (Ji et al., 2017; Sandrini et al., 2016). To explore the mechanism of this difference, a model was developed to simulate whether interspecific differences in carbon absorption capacity determine the response of algal competitive advantage to elevated atmospheric CO_2_ concentration. According to the carbon absorption capacity, algal species can be divided into species with high affinity for both CO_2_ and HCO_3_^−^ (HCHH); species with high affinity for CO_2_ and low affinity for HCO_3_^−^ (HCLH); species with low affinity for CO_2_ and high affinity for HCO_3_^−^ (LCHH); species with low affinity for both CO_2_ and HCO_3_^−^ (LCLH).

Based on previous studies (Anazawa, 2012; Ji et al., 2017; Lindberg & Collins, 2020; Schippers, Mooij, et al., 2004a; Verspagen et al., 2014), 15 equations were used to construct the model. The environmental conditions set by the model were basically consistent with the experimental conditions.

The CO_2_ in atmosphere enters the water through air‐water exchange. The CO_2_ flux across the air–water interface depends on the difference in partial pressure:

(3) $$f_{t}=\left({p\mathrm{CO}}_{2a}-{p\mathrm{CO}}_{2w_{t}}\right)\times k_{0}\times E$$

*f*_*t*_ is the CO_2_ flux per unit area of air–water interface at time *t*; pCO_2*a*_ is the partial pressure of CO_2_ in atmosphere; *p*CO_2*wt*_ is the partial pressure of CO_2_ in water, pCO_2_wt = CO_2*t*_ /*k*_0_, CO_2*t*_ is the dissolved CO_2_ concentration in the medium at time *t*, *k*_0_ is solubility of carbon dioxide gas, i.e. Henry constant; and *E* is the gas change rate.

After CO_2_ enters the medium, the chemical equilibrium which is CO_2_ + H_2_O⇌H_2_CO_3_⇌H^+^+HCO_3_^−^⇌H^+^+CO_3_^2−^ would change, resulting in the decrease of pH in water. Studies have shown that water pH will decrease by about 0.01 units for each increase of 1 Pa of PCO_2_, so water pH is related to the partial pressure of CO_2_ in water:

(4) $${\mathrm{pH}}_{t}={\mathrm{pH}}_{0}-∆{\mathrm{PCO}}_{2w}*B*0.01$$

pH_*t*_ and pH_0_ are pH values at time *t* and in initial time, respectively; ΔPCO_2*w*_ is the change in partial pressure of CO_2_ in water; *B* is the cushion coefficient.

The concentration of total dissolved inorganic carbon (DIC = CO_2_ + HCO_3_^−^ + CO_3_^2−^) in water changes with the amount of CO_2_ entering the water. At the same time, algal growth will absorb CO_2_ and HCO_3_^−^ in water, and algal respiration will release CO_2_. These processes also change the DIC concentration. Therefore, the variation of DIC concentration with time can be expressed as:

(5) $$\frac{d\left[\mathrm{DIC}\right]}{\mathrm{dt}}=\frac{f_{t}}{z}-\sum_{s=1}^{n}\left(u1_{s,t}+u2_{s,t}\right)X_{s,t}+\sum_{s=1}^{n}r_{s,t}X_{s,t}$$

*z* is the depth of water column, *f* division by *z* converts the flux per unit surface area into the corresponding change in DIC concentration; *u*1 and *u*2 are uptake of dissolved CO_2_ and HCO_3_^−^ by the photosynthetic activity of the algae community, respectively (as calculated by Equations 8 and 9); *r* is the respiration rate (as calculated by Equation 11); *X* is population density of algae (as calculated by Equation 13); *s* is the algae species, *n* is the number of species, when *n* = 1, it means that there is only one species, that is, it simulates the situation of monoculture, and *s* = 1; when *n* = 2, it means that the simulated situation is mixture culture, and *s* = 1 or 2.

According to the equilibrium dissociation of DIC (CO_2_ + HCO_3_^−^ + CO_3_^2−^) components, changes in the concentration of dissolved CO_2_ and HCO_3_^−^ are described by:

(6) $${\left[{\mathrm{CO}}_{2}\right]}_{t}=\frac{{{\left[H^{+}\right]}_{t}}^{2}\times {\left[\mathrm{DIC}\right]}_{t}}{{{\left[H^{+}\right]}_{t}}^{2}+k_{1}{\left[H^{+}\right]}_{t}+k_{1}k_{2}}$$

(7) $${\left[{\mathrm{HCO}}_{3}^{-}\right]}_{t}=\frac{k_{1}{\left[H^{+}\right]}_{t}\times {\left[\mathrm{DIC}\right]}_{t}}{{{\left[H^{+}\right]}_{t}}^{2}+k_{1}{\left[H^{+}\right]}_{t}+k_{1}k_{2}}$$

*k*_1_ and *k*_2_ are the equilibrium dissociation constants of CO_2_ and HCO_3_^−^, respectively.

The uptake rate of dissolved CO_2_ and HCO_3_^−^ by the photosynthetic activity of the algae community in Equation 5 depends on the substrate concentration and the affinity and flux rate of species *s* to the substrate (Here, affinity and flux rates are quantified by half‐saturation constant and maximum absorption rate, respectively. Half‐saturation constant are the substrate concentrations required to reach half of the maximum absorption rate. The higher half‐saturation constant is, the worse the substrate capture ability of the binding site on the transporter is, so it is inversely proportional to affinity. The maximum absorption rate is the substrate absorption rate of species when the binding site on the transporter is saturated, and the maximum absorption rate is proportional to the flux rate), as well as the intensity of light and the carbon contents in the cell:

(8) $$u1_{s,t}=\frac{u1_{\max,s}\times {\left[{\mathrm{CO}}_{2}\right]}_{t}}{H1_{s}+{\left[{\mathrm{CO}}_{2}\right]}_{t}}\times P_{t}\times \frac{1-Q_{s,t}}{Q_{\max}}$$

(9) $$u2_{s,t}=\frac{u2_{\max,s}\times {\left[{\mathrm{HCO}}_{3}^{-}\right]}_{t}}{H2_{s}+{\left[{\mathrm{HCO}}_{3}^{-}\right]}_{t}}\times P_{t}\times \frac{1-Q_{s,t}}{Q_{\max}}$$

*u*1_max,*s*_ and *u*2_max,*s*_ are the maximum absorption rate of species *s* to CO_2_ and HCO_3_^−^ respectively; *H*1_*s*_ and *H*2_*s*_ are the half‐saturation constants of species *s* to CO_2_ and HCO_3_^−^ respectively; *P* is the photosynthetic rate; *Q*_*s*_ is the cellular carbon content; and *Q*_max_ is the maximum amount of carbon that can be stored in its cell. The cellular carbon content is proportional to the growth rate and respiration rate:

(10) $$g_{s,t}=g_{\max,s}\times \frac{Q_{s,t}}{Q_{\max}}$$

(11) $$r_{s,t}=r_{\max,s}\times \frac{Q_{s,t}}{Q_{\max}}$$

*g*_*s,t*_ and *r*_*s,t*_ are the growth rate and respiration rate of species s, respectively; *g*_max,*s*_ and *r*_max,*s*_ are the maximum growth rate and the maximum respiration rate of species *s*, respectively. At the same time, the carbon absorption process of algae increases the amount of carbon in cells, and the growth and respiration of algae consumes carbon in cells, and these processes determine the change of cellular carbon content:

(12) $$\frac{dQ_{s}}{\mathrm{dt}}=u1_{s,t}+u2_{s,t}-r_{s,t}-g_{s,t}$$

With the propagation of algae, the population density becomes larger. The change of the population density of algae over time is as follows:

(13) $$\frac{dX_{s}}{\mathrm{dt}}=g_{s,t}\left(1-m\right)X_{s}\left(1-\frac{X_{s}}{C}\right)$$

*m* is the mortality rate, and *C* is the environmental capacity. After the population density in monoculture and pairwise competition experiments are calculated, the competitive ability of each species is calculated by RNE (Equation 2).

The continuous increasing of population density may cause a self‐shading effect that affects light intensity, and the photosynthetic rate at average depth can be expressed as the average of the photosynthetic rate at all depths:

(14) $$P_{t}=\frac{1}{z}\int_{0}^{z}P\left(I{\left(z\right)}_{t}\right)dz$$

*I* is light intensity, and the notation *P*(*I*[*z*]) indicates that the photosynthetic rate is a function of the local light intensity *I*, which in turn is a function of depth *z*. *P*(*I*) and *I*(*z*)_*t*_ are represented by the equations:

(15) $$P\left(I\right)=\frac{P_{\max}I}{\left(P_{\max}/\alpha \right)+I}$$

(16) $$I{\left(z\right)}_{t}=I_{\text{in}}\exp\left(-k_{\mathrm{bg}}z-kX_{t}z\right)$$

where *P*_max_ is the maximum photosynthetic rate; *α* is the slope of the *p*(*I*) curve at *I* = 0; *I*_in_ is the incident light intensity at the top of the column; *K*_*bg*_ is the background turbidity of the medium; and *k* is the specific light attenuation coefficient of an algae cell.

The ten levels of atmospheric CO_2_ concentration were 200, 400, 600, 800, 1000, 1200, 1400, 1600, 1800 and 2000 ppm. The concentration of CO_2_ in the atmosphere is expected to rise from current levels of 380 ppm to 1000 ppm within the next century (Bulling et al., 2010). In addition, CO_2_ in freshwater ecosystems does not only originate from dissolution of atmospheric CO_2_ but also from mineralization of organic carbon obtained from terrestrial sources in the surrounding watershed (Verspagen et al., 2014). Therefore, a large range of CO_2_ concentration level was set, that is, 200–2000 ppm.

As mentioned above, algae species were classified into four kinds based on their affinity and flux rate for CO_2_ and HCO_3_^−^, and affinity and flux rate were quantified by half‐saturation constant and maximum absorption rate, respectively. The corresponding parameter settings of each kind of algae are: HCHH: *H*1 = 1, *H*2 = 30, *u*1_max_ = 0.2, *u*2_max_ = 0.2; HCLH: *H*1 = 1, *H*2 = 1200, *u*1_max_ = 0.2, *u*2_max_ = 0.4; LCHH: *H*1 = 40, *H*2 = 30, *u*1_max_ = 0.4, *u*2_max_ = 0.2; LCLH: *H*1 = 40, *H*2 = 1200, *u*1_max_ = 0.4, *u*2_max_ = 0.4. Since the concentration of HCO_3_^−^ in fresh water is generally much higher than that of CO_2_, the affinity for CO_2_ over HCO_3_^−^ is assumed to be one order of magnitude higher. The values of other performance parameters of algae and environmental conditions are same among algal species (Table 1). The model was run over 1000 time‐steps, such that the algal community stabilized by the end of the run.

**TABLE 1:** Parameter settings in the model

Parameter	Description	Values	Units
*k*_0_	Solubility of CO_2_ gas, Henry's constant	0.375	μmol·L^−1^·pa^−1^
*E*	Gas transfer velocity	2	Dm
*z*	Depth	5	Dm
*k*_1_	Equilibrium dissociation constant of CO_2_	0.43	μmol·L^−1^
*k*_2_	Equilibrium dissociation constant of HCO_3_^−^	5.6 × 10^−5^	μmol·L^−1^
*Q*_max_	Maximum cellular carbon content	1	μmol·L^−1^·cell^−1^
*g*_max_	Maximum growth rate	1	
*r*_max_	Maximum respiration rate	0.2	
m	Mortality	0.4	
*C*	Environmental capacity	10^12^	cells·L^−1^
*I*_in_	Incident light intensity	50	μmol·m^−2^·s^−1^
*k*_bg_	Background turbidity	0.5	dm^−1^
*k*	Specific light attenuation coefficient	10^−6^	dm^−1^

R 3.5.1 was used to run the simulation. An ANOVA followed by Tukey's HSD test was used to test the effects of CO_2_ and species on the RNE values. An ANOVA followed by Tukey's HSD test was used to test the effects of measurement time (length of growth time), interspecific interaction, CO_2_, and species on growth rate. The significance level was set at 0.05. The statistical analyses were performed using SPSS 22.0 (IBM, USA).

## 3 RESULTS

**FIGURE 1:**
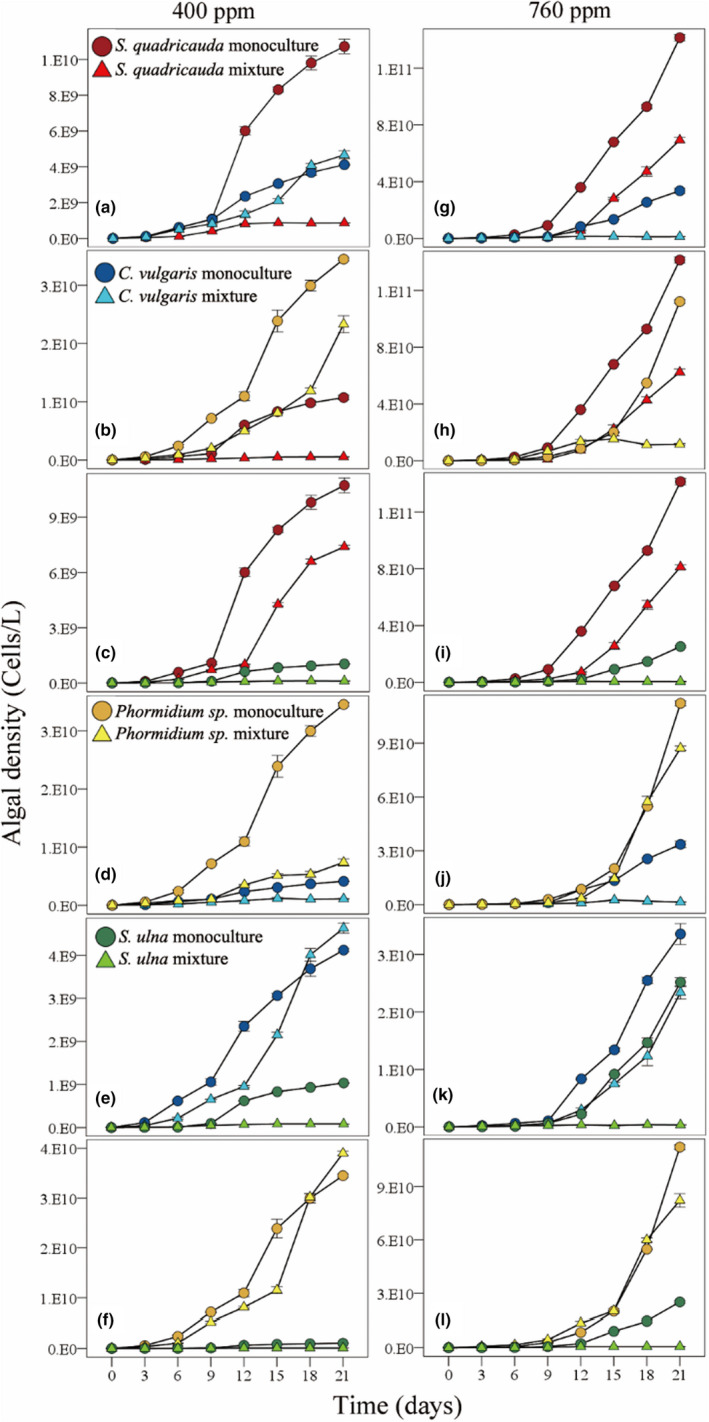
Effects of time, atmospheric CO_2_ concentration, and competition on the density of *Phormidium* sp., *Scenedesmus quadricauda*, *Chlorella vulgaris* and *Synedra ulna* over time in the experiments. Figures (a)‐(f) are the algal density in the pairwise competition experiments when CO_2_ concentration was 400 ppm; figures (g)‐(I) are the algal density in the pairwise experiments when CO_2_ concentration was 760 ppm. Standard errors of three replicates are shown

In the experiments, the cell density of all algal species increased significantly when the atmospheric CO_2_ concentration increased from 400 ppm to 760 ppm (Table S1; Figure 1). At 400 ppm, the algae could be ranked according to cell density: *Phormidium* sp > *C. vulgaris* > *S. quadricauda* > *Synedra ulna*; at 760 ppm, the algae could be ranked according to cell density: *S. quadricauda* > *Phormidium* sp > *C. vulgaris* > *S. ulna* (Figure 1).

According to the calculation method of RNE, the RNE value of *S. quadricauda* reflected the potential decrease in cell density when *S. quadricauda* cultured with C*. vulgaris*, *Phormidium* sp. and *S. ulna* comparing to *S. quadricauda* cultured alone, and the same behavior mostly occurred when the other three species compete in pairs, except for the potential increase in cell density when *C. vulgaris* cultured with *S. quadricauda* and *S. ulna*, and *Phormidium* sp. cultured with *S. ulna* at 400 ppm.

**FIGURE 2:**
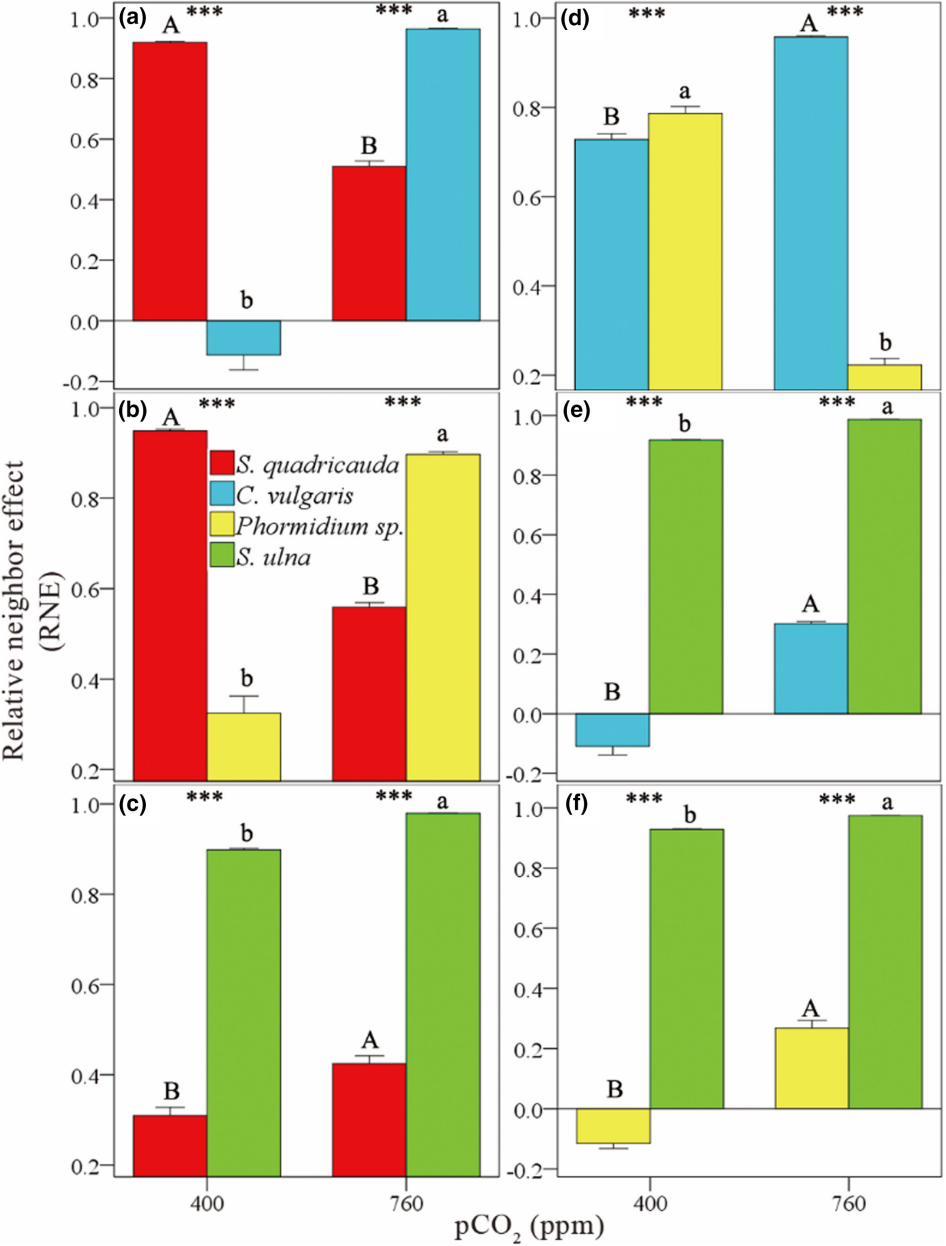
Effects of atmospheric CO_2_ concentration on interactions between *S. quadricauda* and *C. vulgaris* (a), *S. quadricauda* and *Phormidium* sp. (b), *S. quadricauda* and *S. ulna* (c), *C. vulgaris* and *Phormidium* sp. (d), *C. vulgaris* and *S. ulna* (e), *Phormidium* sp. and *S. ulna* (f) in the experiments. The mean interspecific relative neighbor effects (RNE) on total density are shown. Capital and lowercase letters indicate significant differences in RNE of the two species along the CO_2_ gradient. Asterisks indicate significant differences in RNE between the two species (*p < .05, **p < .01, ***p < .001, NS, not significant). Standard errors of three replicates are shown

A positive value of RNE reflected the decrease cell density and therefore indicated the interspecific competition; a negative value of RNE reflected the increase cell density and therefore indicated the interspecific facilitation. The RNE values of *S. quadricauda* in mixture with other three species were all positive along the CO_2_ gradient, and the same pattern was observed for *S. ulna* in mixture with other three species, *C. vulgaris* in mixture with *Phormidium* sp., *Phormidium* sp. in mixture with *S. quadricauda* and *C. vulgaris*, respectively (Figure 2), indicating that the interaction between *S. quadricauda* and other three species, *S. ulna* and other three species, *C. vulgaris* and *Phormidium sp*., *Phormidium* sp. and *S. quadricauda*, *Phormidium* sp. and *C. vulgaris* were interspecific competition. When *C. vulgaris* in mixture with *S. quadricauda* and *S. ulna*, respectively, and *Phormidium* sp. in mixture with *S. ulna*, the RNE values changed from negative to positive along the CO_2_ gradient (Figure 2), indicating that the interaction between *C. vulgaris* and *S. quadricauda*, *C. vulgaris* and *S. ulna*, and *Phormidium* sp. and *S. ulna* was interspecific facilitation.

Differences in RNE among species indicated that at low atmospheric CO_2_ concentration (400 ppm), the algae were ranked according to the competitive ability: *C. vulgaris* > *Phormidium* sp. > *S. quadricauda* > *Synedra ulna* (Figure 2). When the CO_2_ concentration increased to 760 ppm, the algae were ranked according to their competitive ability: *S. quadricauda* > *Phormidium* sp. > *C. vulgaris* > *S. ulna* (Table 2; Figure 2).

**TABLE 2:** Summary of ANOVA of the effects of species and CO_2_ on the relative neighbor effect (RNE) of *Scenedesmus quadricauda*, *Chlorella vulgaris*, *Phormidium* sp. and *Synedra ulna*

Source	Species			CO_2_			Species × CO_2_		
	*df*	*F*	*p*	*df*	*F*	*p*	*df*	*F*	*p*
*S. quadricauda* and *C. vulgaris*	1	116.42	<.001	1	154.40	<.001	1	768.08	<.001
*S. quadricauda* and *Phormidium* sp.	1	51.72	<.001	1	20.86	<.01	1	580.41	<.001
*S. quadricauda* and *S. ulna*	1	2103.06	<.001	1	61.87	<.001	1	1.88	.207
*C. vulgaris* and *Phormidium sp*.	1	708.64	<.001	1	172.27	<.001	1	974.54	<.001
*C. vulgaris* and *S. ulna*	1	3282.66	<.001	1	257.64	<.001	1	131.23	<.001
*Phormidium* sp. and *S. ulna*	1	3167.79	<.001	1	189.86	<.001	1	117.47	<.001

*Note*: *p* < .05 is taken to be significant.

**FIGURE 3:**
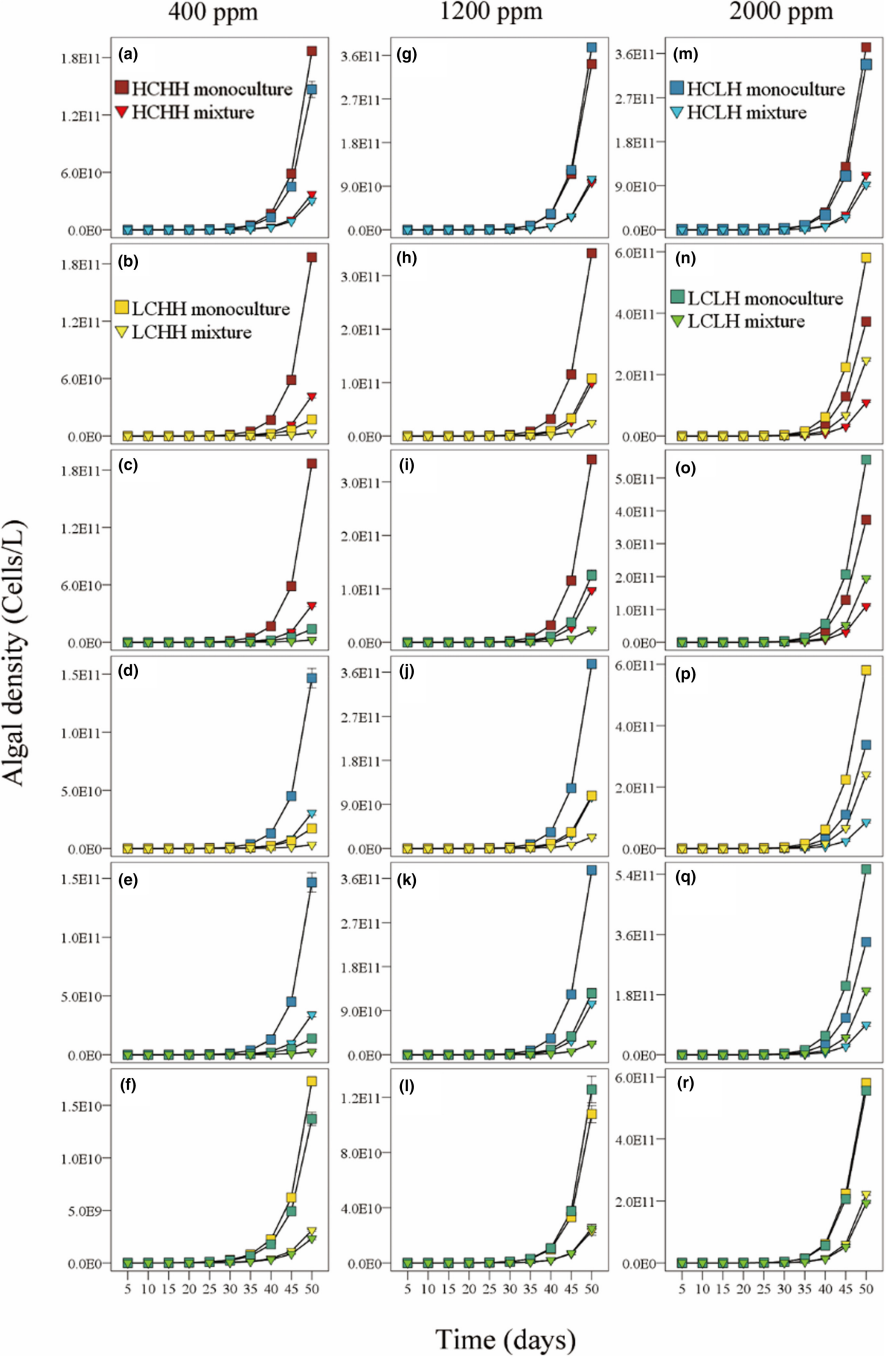
Effects of time, atmospheric CO_2_ concentration, and competition on the density of the species with high affinity for both CO_2_ and HCO_3_^−^ (HCHH), the species with high affinity for CO_2_ and low affinity for HCO_3_^−^ (HCLH), the species with low affinity for CO_2_ and high affinity for HCO_3_^−^ (LCHH) and the species with low affinity for both CO_2_ and HCO_3_^−^ (LCLH) over time in the model. Figures (a)‐(f) are the algal density in the pairwise competition experiments when CO_2_ concentration was 400 ppm; figures (g)‐(I) are the algal density in the pairwise experiments when CO_2_ concentration was 1200 ppm; figures (m)‐(r) are the algal density in the pairwise experiments when CO_2_ concentration was 2000 ppm. Standard errors of five replicates are shown

The simulation results showed that the cell density of all four algae increased significantly with the increase of CO_2_ concentration (Table S2; Figure 3). At 400 ppm, the algae could be ranked according to cell density: HCHH > HCLH > LCHH > LCLH; at 1200 ppm, the algae could be ranked according to cell density: HCLH > HCHH > LCLH > LCHH; at 2000 ppm, the algae could be ranked according to cell density: LCHH > LCLH > HCHH > HCLH (Figure 3).

**FIGURE 4:**
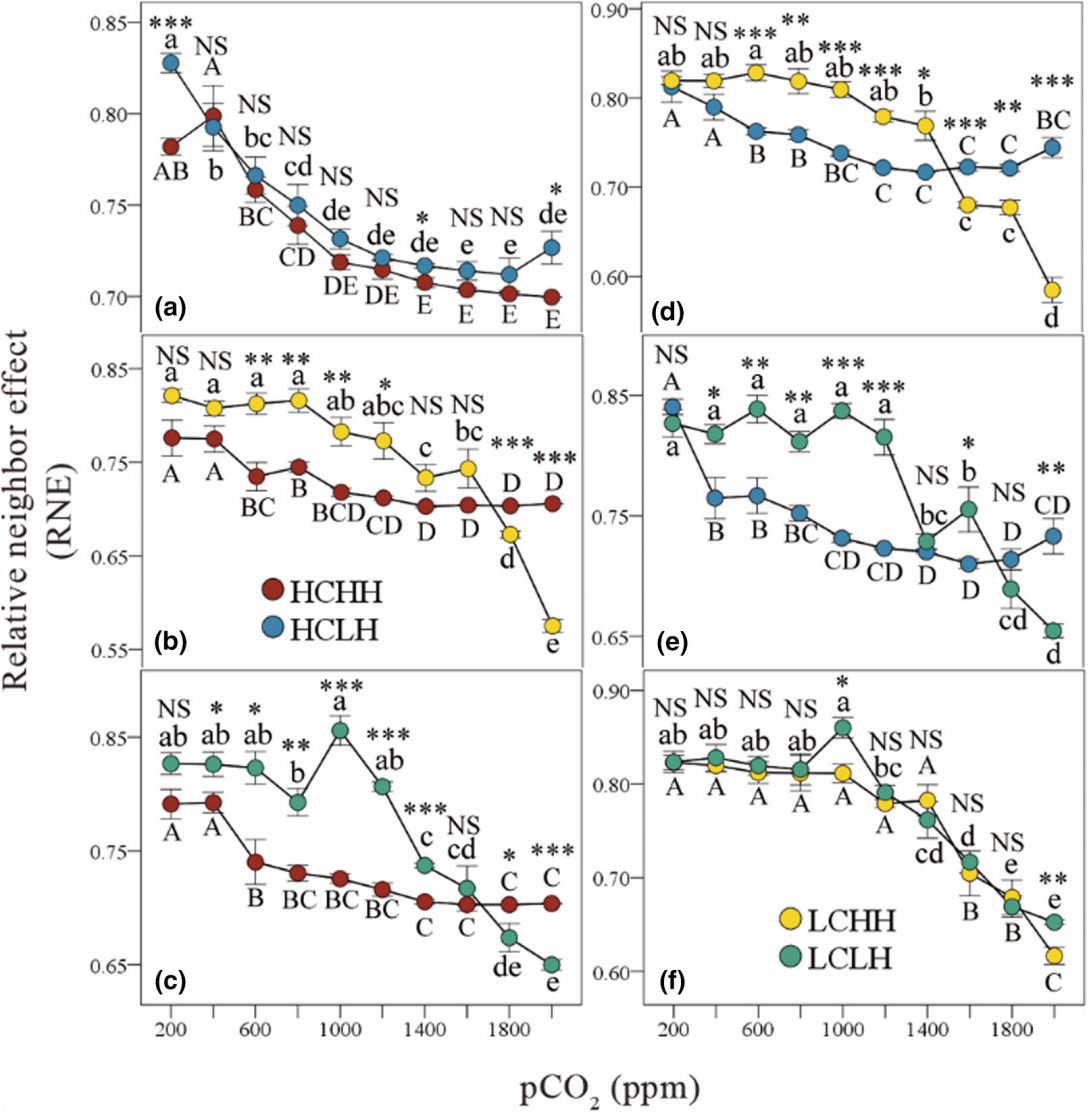
Effects of atmospheric CO_2_ concentration on interactions between HCHH and HCLH (a), HCHH and LCHH (b), HCHH and LCLH (c), HCLH and LCHH (d), HCLH and LCLH (e), LCHH and LCLH (f) in the model. The mean interspecific relative neighbor effects (RNE) on total density are shown. Capital and lowercase letters indicate significant differences in RNE of the two species along the CO_2_ gradient. Asterisks indicate significant differences in RNE between the two species (*p < .05, **p < .01, ***p < .001, NS, not significant). Standard errors of five replicates are shown. HCHH refers to the species with high affinity for both CO_2_ and HCO_3_^−^; HCLH refers to the species with high affinity for CO_2_ and low affinity for HCO_3_^−^; LCHH refers to the species with low affinity for CO_2_ and high affinity for HCO_3_^−^; LCLH refers to the species with low affinity for both CO_2_ and HCO_3_^−^

According to the calculation method of RNE, the RNE values of the HCHH species reflected the potential decrease in cell density when the HCHH species grew with other three species comparing to HCHH species grew alone, and the same behavior occurred when the other three species competed in pairs. The RNE values of the four species growing in pairs were all positive on the CO_2_ gradient (Figure 4), indicating that the interaction of the four species growing in pairs were interspecific competition. The differences in RNE among species showed that when the CO_2_ concentration was low (200–1600 ppm), the algae were ranked according to the competitive ability: HCHH >HCLH >LCHH >LCLH; when the CO_2_ concentration was high (1800–2000 ppm), the algae were ranked according to the competitive ability: LCHH > LCLH > HCHH > HCLH (Table 3; Figure 4).

**TABLE 3:** Summary of ANOVA of the effects of species and CO_2_ on the relative neighbor effect (RNE) of the species with high affinity for both CO_2_ and HCO_3_^−^ (HCHH), the species with high affinity for CO_2_ and low affinity for HCO_3_^−^ (HCLH), the species with low affinity for CO_2_ and high affinity for HCO_3_^−^ (LCHH) and the species with low affinity for both CO_2_ and HCO_3_^−^ (LCLH) in the model

Source	Species			CO_2_			Species × CO_2_		
	*df*	*F*	*p*	*df*	*F*	*p*	*df*	*F*	*p*
HCHH and HCLH	1	8.55	<.01	1	25.25	<.001	1	0.91	.522
HCHH and LCHH	1	21.90	<.001	1	31.74	<.01	1	12.75	<.001
HCHH and LCLH	1	43.47	<.001	1	26.57	<.001	1	8.45	.207
HCLH and LCHH	1	2.71	.104	1	30.77	<.001	1	15.73	<.001
HCLH and LCLH	1	34.28	<.001	1	28.51	<.001	1	11.20	<.001
LCHH and LCLH	1	1.59	.211	1	32.68	<.001	1	0.66	.743

*Note*: *p* < .05 is taken to be significant.

**FIGURE 5:**
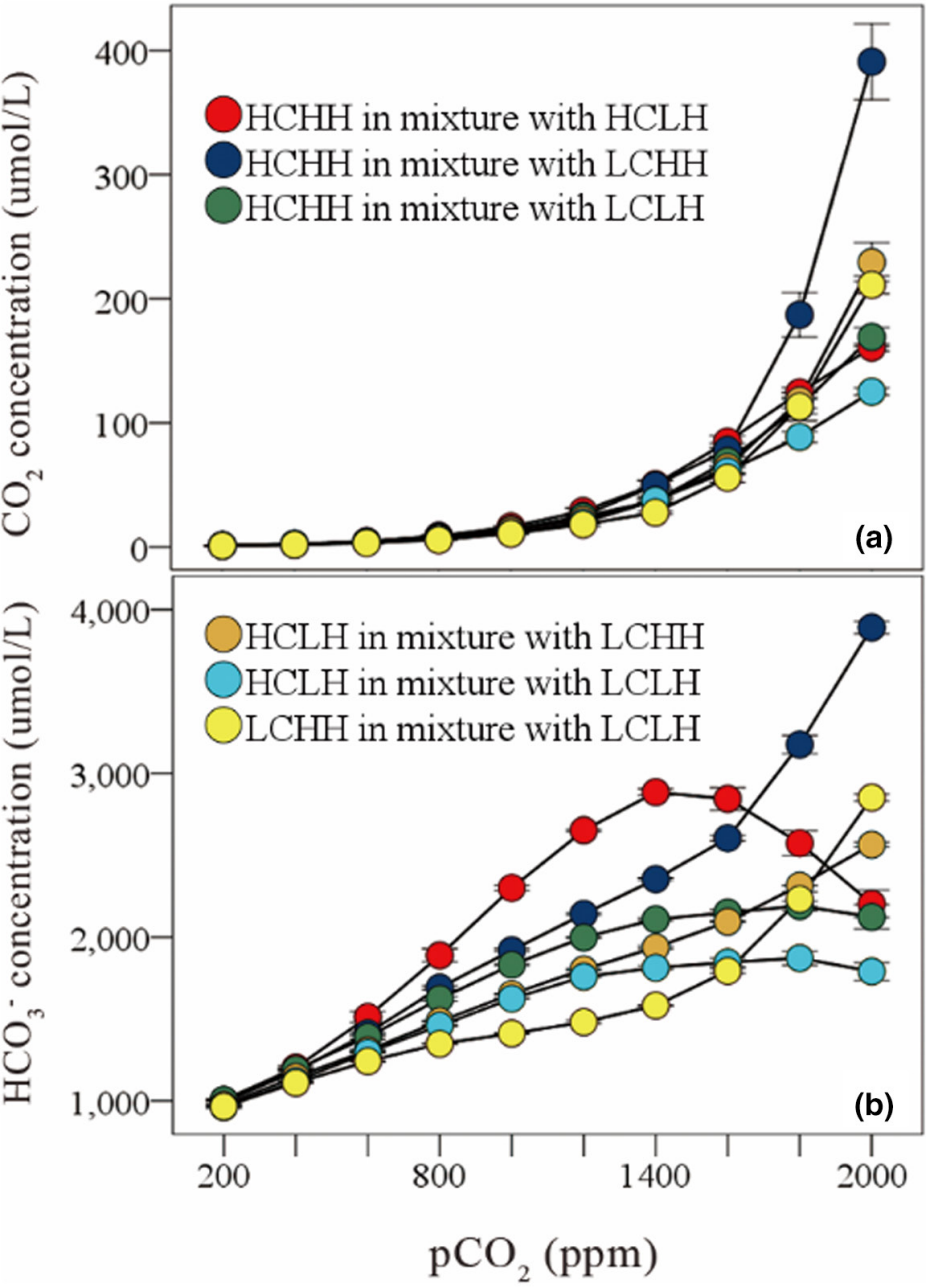
Effects of atmospheric CO_2_ concentration on CO_2_ concentration (a) and HCO_3_^−^ concentration (b) in pairwise mixed simulation. HCHH refers to the species with high affinity for both CO_2_ and HCO_3_^−^; HCLH refers to the species with high affinity for CO_2_ and low affinity for HCO_3_^−^; LCHH refers to the species with low affinity for CO_2_ and high affinity for HCO_3_^−^; LCLH refers to the species with low affinity for both CO_2_ and HCO_3_^−^

With the increase of atmospheric CO_2_ concentration, the CO_2_ concentration in water increased significantly; when the HCHH, HCLH and LCLH species were mixed in pairs, the HCO_3_^−^ concentration first increased and then decreased, and when the LCHH species and other species are mixed in pairs, respectively, the HCO_3_^−^ concentration increased significantly (Table 4; Figure 5).

**TABLE 4:** Summary of ANOVA of the effects of species and CO_2_ on environmental factors in the model

Source	CO_2_			HCO_3_^−^		
	*df*	*F*	*p*	*df*	*F*	*p*
Species	5	56.20	<.001	5	873.15	<.001
CO_2_	9	857.27	<.001	9	2392.82	<.001
Species × CO_2_	45	27.05	<.001	45	127.06	<.001

*Note*: *p* < .05 is taken to be significant.

## 4 DISCUSSION

Our study showed that the competitive ability of algae changed differently when CO_2_ increased from 400 to 760 ppm, and the different changes of competitiveness between algae species along the gradient of atmospheric CO_2_ concentration was due to the interspecific differences in affinity and flux rate for CO_2_ and HCO_3_^−^. These results provide an important perspective for understanding and predicting the changes of population dynamics and community composition of algae under the background of increasing global atmospheric CO_2_.

The results of experiments showed that when the CO_2_ concentration increases from 400 to 760 ppm, the competitiveness of *S. quadricauda* increased, the competitiveness of *Phormidium* sp. and *C. vulgaris* decreased, and the competitive dominant species changed from *C. vulgaris* to *S. quadricauda*. Thus, the competitive ability of different algae species responded differently to the increase of atmospheric CO_2_ concentration, even though they belonged to the same taxa (both *C. vulgaris* and *S. quadricauda* belonged to green algae). Other ecologists have also shown that the competitiveness of different algal species within the same taxa varies differently along atmospheric CO_2_ level gradients (Ji et al., 2017; Sandrini et al., 2016).

Our simulation results showed that the reason for this difference of competitiveness is that algae have different absorption capacity for CO_2_ and HCO_3_^−^, that is, different affinity and flux rates for CO_2_ and HCO_3_^−^. Affinity and flux rate are the capture capacity and transport capacity of substrate, respectively, which are inversely proportional to each other. Low resource concentration is beneficial to the growth and reproduction of algae with high affinity and high resource concentration is beneficial to the growth and reproduction of algae with high flux rate. According to the carbon absorption capacity of algae, algae are divided into four types: HCHH species with high affinity for both CO_2_ and HCO_3_^−^; HCLH species with high affinity for CO_2_ and low affinity for HCO_3_^−^; LCHH species with low affinity for CO_2_ and high affinity for HCO_3_^−^; LCLH species with low affinity for both CO_2_ and HCO_3_^−^.

The increase of atmospheric CO_2_ concentration affected the competitiveness of algae with different carbon absorption capacity by affecting the carbon balance in freshwater ecosystem. When the atmospheric CO_2_ concentration is low, both the CO_2_ and HCO_3_^−^ in water are low, then the species with high affinity for both CO_2_ and HCO_3_^−^ had the highest competitiveness. When atmospheric CO_2_ increases, CO_2_ in water increases rapidly, while HCO_3_^−^ increases slowly or even decreases due to the decrease of pH. On this condition, the species with low affinity for CO_2_ and high affinity for HCO_3_^−^ would be dominant. Thus, with the increase of atmospheric CO_2_ concentration, the dominant species changed from HCHH species to LCHH species.

Ji et al. investigated the competitive relationship between a harmful cyanobacteria and three green algae at low and high CO_2_ concentrations. The results showed that two of the green algae were competitively superior to the cyanobacteria at low CO_2_, whereas the competitive ability of cyanobacteria increased compared to the green algae at high CO_2_ (Ji et al., 2017). Sandrini et al. showed that the increased CO_2_ availability will be beneficial for the low affinity but high flux bicarbonate absorption system, and cyanobacteria with this absorption system are likely to become the main component of cyanobacteria bloom in the future (Sandrini et al., 2016). These results imply that the carbon absorption capacity is the root cause for interspecific differences in competitiveness of algae.

Since cyanobacteria bloom has become a major water quality problem in many eutrophic lakes around the world, previous studies mostly focused on the change of competitive advantage between cyanobacteria and eukaryotic algae (Bestion et al., 2018; Huisman et al., 2018; Ji et al., 2017; Ma et al., 2019). The traditional view is that rising CO_2_ levels will particularly benefit eukaryotic phytoplankton species rather than cyanobacteria because cyanobacteria have developed an efficient CO_2_ concentration mechanism (CCM) to adapt to the low CO_2_ environment (Badger & Price, 2003; Huisman et al., 2018; Ma et al., 2019; Wolf et al., 2019). However, with the in‐depth study, researchers found that eukaryotic algae also have a complex CCM mechanism to adapt to low CO_2_ concentration (Giordano et al., 2005; Ji et al., 2017). In addition, recent studies have founded that some cyanobacteria have low affinity but high flux bicarbonate absorption system to adapt to the high CO_2_ concentration (Sandrini et al., 2014, 2016; Visser et al., 2016). Thus, the carbon absorption capacity of algae is an important attribute to predict its response to elevated CO_2_.

In addition to the response of algal growth to atmospheric CO_2_ concentration, our model also includes the influence of the photosynthesis and respiration of algae on the change of inorganic carbon concentration in water (Equation 5). The algal communities may influence CO_2_ emissions into the atmosphere and thus feedback on the ongoing and future climate change (Lewington‐Pearce et al., 2020). However, the interaction between algal growth and CO_2_ concentration has not been fully studied. Therefore, the importance of aquatic plants in the global carbon cycle should be considered in future studies on the response of aquatic plants to climate change, to predict the trend of future climate change and the response mechanism of growth of aquatic plants more comprehensively.

## 5 CONCLUSION

This study highlights the importance of carbon absorption capacity in understanding, predicting and regulating population dynamics and community composition of algae. According to the carbon absorption capacity, algae species can be classified as HCHH, HCLH, LCHH and LCLH species. Whether cyanobacteria or eukaryotes, HCHH species should be paid more attention at low CO_2_ levels; while LCHH species should be paid more attention at high CO_2_ levels. These results help understanding algal population dynamics and community composition along environmental gradients, predicting bloom causing species under the background of increasing global atmospheric CO_2_, and providing an important basis for maintaining the health of aquatic ecosystem.

## AUTHOR CONTRIBUTIONS

**Qing Shi Zhou:** Conceptualization (equal); formal analysis (equal); investigation (equal); methodology (equal); software (equal); validation (equal); writing – original draft (equal); writing – review and editing (equal). **Yang Gao:** Data curation (equal); funding acquisition (equal); investigation (equal); supervision (equal); validation (equal); visualization (equal); writing – original draft (equal). **Jing Ming Hou:** Data curation (equal); project administration (equal); resources (equal); software (equal); supervision (equal); validation (equal); visualization (equal). **Tian Wang:** Conceptualization (equal); formal analysis (equal); methodology (equal); software (equal); visualization (equal). **Long Tang:** Conceptualization (equal); formal analysis (equal); funding acquisition (equal); project administration (equal); validation (equal); writing – original draft (equal); writing – review and editing (equal).

## CONFLICT OF INTEREST

The authors declare no competing interests.

## Supporting information


Tables S1 and S2

## Data Availability

Data available from the Dryad Digital Repository (https://doi.org/10.5061/dryad.d7wm37q40).
