# Supplementary material for: Preference of carbon absorption determines the competitive ability of algae along atmospheric CO2 concentration
Source: Ecol Evol. 2022 Jul 11;12(7):e9079. doi: 10.1002/ece3.9079 (PMC9274100; doi:10.1002/ece3.9079)
Supplement: Supplementary file 1 — Tables S1 and S2 [file ECE3-12-e9079-s001.docx]

**Table S1** Summary of repeated measures ANOVA of the effects of CO_2_, species and competition on the cell density of *Scenedesmus quadricauda*, *Chlorella vulgaris*, *Phormidium sp.* and *Synedra ulna*.

| **Source** | ***S. quadricauda***  **and *C. vulgaris*** | | | ***S. quadricauda***  ***and Phormidium sp.*** | | | ***S. quadricauda***  **and *S. ulna*** | | | ***C. vulgaris***  **and *Phormidium sp.*** | | | ***C. vulgaris***  **and *S. ulna*** | | |  | | |
| --- | --- | --- | --- | --- | --- | --- | --- | --- | --- | --- | --- | --- | --- | --- | --- | --- | --- | --- |
|  | df | *F* | *p* | df | *F* | *p* | df | *F* | *p* | df | *F* | *p* | df | *F* | *p* | df | *F* | *p* |
| Between subjects |  |  |  |  |  |  |  |  |  |  |  |  |  |  |  |  |  |  |
| CO_2_ | 1 | 22758.96 | <0.001 | 1 | 10921.14 | <0.001 | 1 | 11496.16 | <0.001 | 1 | 5008.17 | <0.001 | 1 | 2494.34 | <0.001 | 1 | 1152.65 | <0.001 |
| Species | 1 | 15022.35 | <0.001 | 1 | 407.74 | <0.001 | 1 | 11662.57 | <0.001 | 1 | 8259.59 | <0.001 | 1 | 1069.19 | <0.001 | 1 | 6161.90 | <0.001 |
| CO_2_ × species | 1 | 13380.47 | <0.001 | 1 | 4477.55 | <0.001 | 1 | 7408.88 | <0.001 | 1 | 1419.32 | <0.001 | 1 | 293.35 | <0.001 | 1 | 351.04 | <0.001 |
| Competition | 1 | 7917.39 | <0.001 | 1 | 7246.22 | <0.001 | 1 | 2623.56 | <0.001 | 1 | 2349.48 | <0.001 | 1 | 893.20 | <0.001 | 1 | 150.11 | <0.001 |
| CO_2_ × competition | 1 | 4807.12 | <0.001 | 1 | 2486.31 | <0.001 | 1 | 1862.65 | <0.001 | 1 | 11.23 | <0.01 | 1 | 691.50 | <0.001 | 1 | 51.17 | <0.001 |
| Species × competition | 1 | 1971.38 | <0.001 | 1 | 96.96 | <0.001 | 1 | 875.29 | <0.001 | 1 | 70.10 | <0.001 | 1 | 25.27 | <0.001 | 1 | 10.60 | <0.01 |
| CO_2_ × species × competition | 1 | 695.54 | <0.001 | 1 | 354.39 | <0.001 | 1 | 578.72 | <0.001 | 1 | 746.17 | <0.001 | 1 | 20.40 | <0.001 | 1 | 41.95 | <0.001 |
| Within subjects |  |  |  |  |  |  |  |  |  |  |  |  |  |  |  |  |  |  |
| Time | 7 | 2836.96 | <0.001 | 7 | 3752.70 | <0.001 | 7 | 2902.52 | <0.001 | 7 | 3573.98 | <0.001 | 7 | 979.86 | <0.001 | 7 | 3568.85 | <0.001 |
| Time × CO_2_ | 7 | 2006.32 | <0.001 | 7 | 1564.98 | <0.001 | 7 | 2071.06 | <0.001 | 7 | 1635.91 | <0.001 | 7 | 599.46 | <0.001 | 7 | 863.93 | <0.001 |
| Time × species | 7 | 1274.09 | <0.001 | 7 | 60.71 | <0.001 | 7 | 1874.39 | <0.001 | 7 | 1791.91 | <0.001 | 7 | 183.74 | <0.001 | 7 | 2386.83 | <0.001 |
| Time × CO_2_ × species | 7 | 1172.85 | <0.001 | 7 | 463.26 | <0.001 | 7 | 1287.48 | <0.001 | 7 | 778.41 | <0.001 | 7 | 68.83 | <0.001 | 7 | 392.28 | <0.001 |
| Time × competition | 7 | 520.07 | <0.001 | 7 | 949.81 | <0.001 | 7 | 329.72 | <0.001 | 7 | 316.49 | <0.001 | 7 | 162.20 | <0.001 | 7 | 100.50 | <0.001 |
| Time × CO_2_ × competition | 7 | 345.20 | <0.001 | 7 | 558.69 | <0.001 | 7 | 257.64 | <0.001 | 7 | 38.22 | <0.001 | 7 | 157.25 | <0.001 | 7 | 141.59 | <0.001 |
| Time × species × competition | 7 | 102.02 | <0.001 | 7 | 62.89 | <0.001 | 7 | 84.04 | <0.001 | 7 | 16.48 | <0.001 | 7 | 31.73 | <0.001 | 7 | 16.73 | <0.001 |
| Time × CO_2_ × species × competition | 7 | 34.36 | <0.001 | 7 | 83.95 | <0.001 | 7 | 56.85 | <0.001 | 7 | 105.13 | <0.001 | 7 | 20.19 | <0.001 | 7 | 31.74 | <0.001 |

*Note*: *p*< 0.05 is taken to be significant

**Table S2** Summary of repeated measures ANOVA of the effects of CO_2_, species and competition on the cell density of the species with high affinity for both CO_2_ and HCO_3_^−^ (HCHH), the species with high affinity for CO_2_ and low affinity for HCO_3_^−^ (HCLH), the species with low affinity for CO_2_ and high affinity for HCO_3_^−^ (LCHH) and the species with low affinity for both CO_2_ and HCO_3_^−^ (LCLH) in the model.

| **Source** | **HCHH**  **and HCLH** | | | **HCHH**  **and LCHH** | | | **HCHH**  **and LCLH** | | | **HCLH**  **and LCHH** | | | **HCLH**  **and LCLH** | | | **LCHH**  **and LCLH** | | |
| --- | --- | --- | --- | --- | --- | --- | --- | --- | --- | --- | --- | --- | --- | --- | --- | --- | --- | --- |
|  | df | *F* | *p* | df | *F* | *p* | df | *F* | *p* | df | *F* | *p* | df | *F* | *p* | df | *F* | *p* |
| Between subjects |  |  |  |  |  |  |  |  |  |  |  |  |  |  |  |  |  |  |
| CO_2_ | 9 | 1219.20 | <0.001 | 9 | 1266.87 | <0.001 | 9 | 1452.28 | <0.001 | 9 | 1207.81 | <0.001 | 9 | 1226.77 | <0.001 | 9 | 2023.28 | <0.001 |
| Species | 1 | 1.17 | 0.282 | 1 | 1178.44 | <0.001 | 1 | 1860.77 | <0.001 | 1 | 1052.47 | <0.01 | 1 | 1577.18 | <0.001 | 1 | 6.56 | <0.05 |
| CO_2_ × species | 9 | 21.66 | <0.001 | 9 | 429.12 | <0.001 | 9 | 410.32 | <0.001 | 9 | 503.59 | <0.001 | 9 | 430.29 | <0.001 | 9 | 6.53 | <0.001 |
| Competition | 1 | 33293.74 | <0.001 | 1 | 10076.16 | <0.001 | 1 | 13167.31 | <0.001 | 1 | 10025.78 | <0.001 | 1 | 11624.84 | <0.001 | 1 | 4815.21 | <0.001 |
| CO_2_ × competition | 9 | 339.46 | <0.001 | 9 | 319.65 | <0.001 | 9 | 399.33 | <0.001 | 9 | 319.43 | <0.001 | 9 | 364.17 | <0.001 | 9 | 537.59 | <0.001 |
| Species × competition | 1 | 1.22 | 0.271 | 1 | 529.24 | <0.001 | 1 | 701.03 | <0.001 | 1 | 577.06 | <0.01 | 1 | 616.46 | <0.001 | 1 | 0.43 | 0.512 |
| CO_2_ × species × competition | 9 | 8.02 | <0.001 | 9 | 95.24 | <0.001 | 9 | 109.27 | <0.001 | 9 | 109.43 | <0.01 | 9 | 117.36 | <0.001 | 9 | 2.44 | <0.05 |
| Within subjects |  |  |  |  |  |  |  |  |  |  |  |  |  |  |  |  |  |  |
| Time | 9 | 95619.55 | <0.001 | 9 | 32070.44 | <0.001 | 9 | 40520.02 | <0.001 | 9 | 31169.77 | <0.001 | 9 | 34344.47 | <0.001 | 9 | 15766.14 | <0.001 |
| Time × CO_2_ | 81 | 1226.80 | <0.001 | 81 | 1279.62 | <0.001 | 81 | 1492.24 | <0.001 | 81 | 1241.74 | <0.001 | 81 | 1263.20 | <0.001 | 81 | 2108.99 | <0.001 |
| Time × species | 9 | 2.50 | <0.01 | 9 | 1249.43 | <0.001 | 9 | 1898.11 | <0.001 | 9 | 1193.21 | <0.001 | 9 | 1686.61 | <0.001 | 9 | 4.35 | <0.001 |
| Time × CO_2_ × species | 81 | 23.08 | <0.001 | 81 | 433.45 | <0.001 | 81 | 425.20 | <0.001 | 81 | 509.73 | <0.001 | 81 | 436.66 | <0.001 | 81 | 5.88 | <0.001 |
| Time × competition | 9 | 31719.74 | <0.001 | 9 | 9593.24 | <0.001 | 9 | 12751.94 | <0.001 | 9 | 9773.69 | <0.001 | 9 | 11355.78 | <0.001 | 9 | 4670.90 | <0.001 |
| Time × CO_2_ × competition | 81 | 309.77 | <0.001 | 81 | 288.00 | <0.001 | 81 | 369.55 | <0.001 | 81 | 296.38 | <0.001 | 81 | 343.14 | <0.001 | 81 | 502.65 | <0.001 |
| Time × species × competition | 9 | 6.10 | <0.001 | 9 | 552.30 | <0.001 | 9 | 690.24 | <0.001 | 9 | 647.35 | <0.001 | 9 | 640.89 | <0.001 | 9 | 0.45 | 0.907 |
| Time × CO_2_ × species × competition | 81 | 8.07 | <0.001 | 81 | 85.13 | <0.001 | 81 | 102.00 | <0.001 | 81 | 96.34 | <0.001 | 81 | 106.70 | <0.001 | 81 | 2.16 | <0.001 |

*Note*: *p*< 0.05 is taken to be significant
